# Laparoscopic Nephrectomy in Patients with Previous Abdominal Surgery

**DOI:** 10.7759/cureus.6991

**Published:** 2020-02-14

**Authors:** Mohamed O Breish, Danielle Whiting, Seshadri Sriprasad

**Affiliations:** 1 Urology, Darent Valley Hospital, Dartford, GBR

**Keywords:** laparoscopic nephrectomy, abdominal adhesion, previous abdominal surgery, complications of nephrectomy

## Abstract

Laparoscopic nephrectomy is a minimally invasive procedure that provides significant benefits to the patient, such as reduced analgesic requirements and shorter recovery time. While the popularity of laparoscopy has grown substantially, there are associated risks of injury to the blood vessels and/or viscera during the insertion of the laparoscopic ports. Such injuries can lead to a significant increase in mortality rates. Patients who have had previous abdominal surgery have a higher risk of adhesions; this has been shown to increase the risk of complications from port placement. Consequently, previous abdominal surgery was viewed as a relative contraindication to laparoscopic surgery. However, studies have demonstrated the advantages of laparoscopic surgery over an open radical approach; hence, previous abdominal surgery is no longer viewed as a contraindication. Here, we describe the case of a 62-year-old man who presented with an incidental finding of right renal cell carcinoma (RCC). We performed a radical nephrectomy on this patient who had undergone multiple previous abdominal surgeries. During this procedure, a small bowel injury occurred. Herein, we review the available evidence and describe the risk factors and techniques to avoid injury from laparoscopic port-site placement in patients undergoing nephrectomy with a history of previous abdominal surgery.

## Introduction

Laparoscopic nephrectomy is a minimally invasive approach that demonstrates comparable oncological outcomes to traditional open nephrectomy and provides significant benefits to the patient, including a reduced analgesic requirement and shorter recovery time [1]. The popularity of laparoscopy has rapidly increased; it is now the most performed technique among radical nephrectomies in the United Kingdom (UK) [2].

However, during laparoscopic surgery, there is a risk of injury to the blood vessels and/or viscera (predominantly bowel) primarily during the insertion of the laparoscopic ports. An analysis of injuries from port placement found that when an injury occurred, there was a significant associated mortality rate of 7.8% [3]. Some studies have demonstrated that the risk of complications from laparoscopic port placement increased in patients with previous abdominal surgery, particularly laparotomy, as a result of adhesions [4]. Consequently, previous abdominal surgery was viewed as a relative contraindication to laparoscopic surgery. However, this posed a problem for a considerable number of patients. At a single center in Baltimore, 48% of the patients requiring urological procedures had a history of previous abdominal surgery [5]. With the clear patient advantages of laparoscopic over an open radical nephrectomy, previous abdominal surgery is no longer viewed as a contraindication. However, the surgeon must undertake careful planning to reduce the risk of complications.

Here, we describe a case of radical nephrectomy in a patient with multiple previous abdominal surgeries in whom a small bowel injury occurred while undergoing the procedure. We also review the available evidence and describe risk factors and techniques to reduce the risk of injury during laparoscopic port-site placement in patients undergoing nephrectomy with a history of abdominal surgery.

## Case presentation

**Figure 1:**
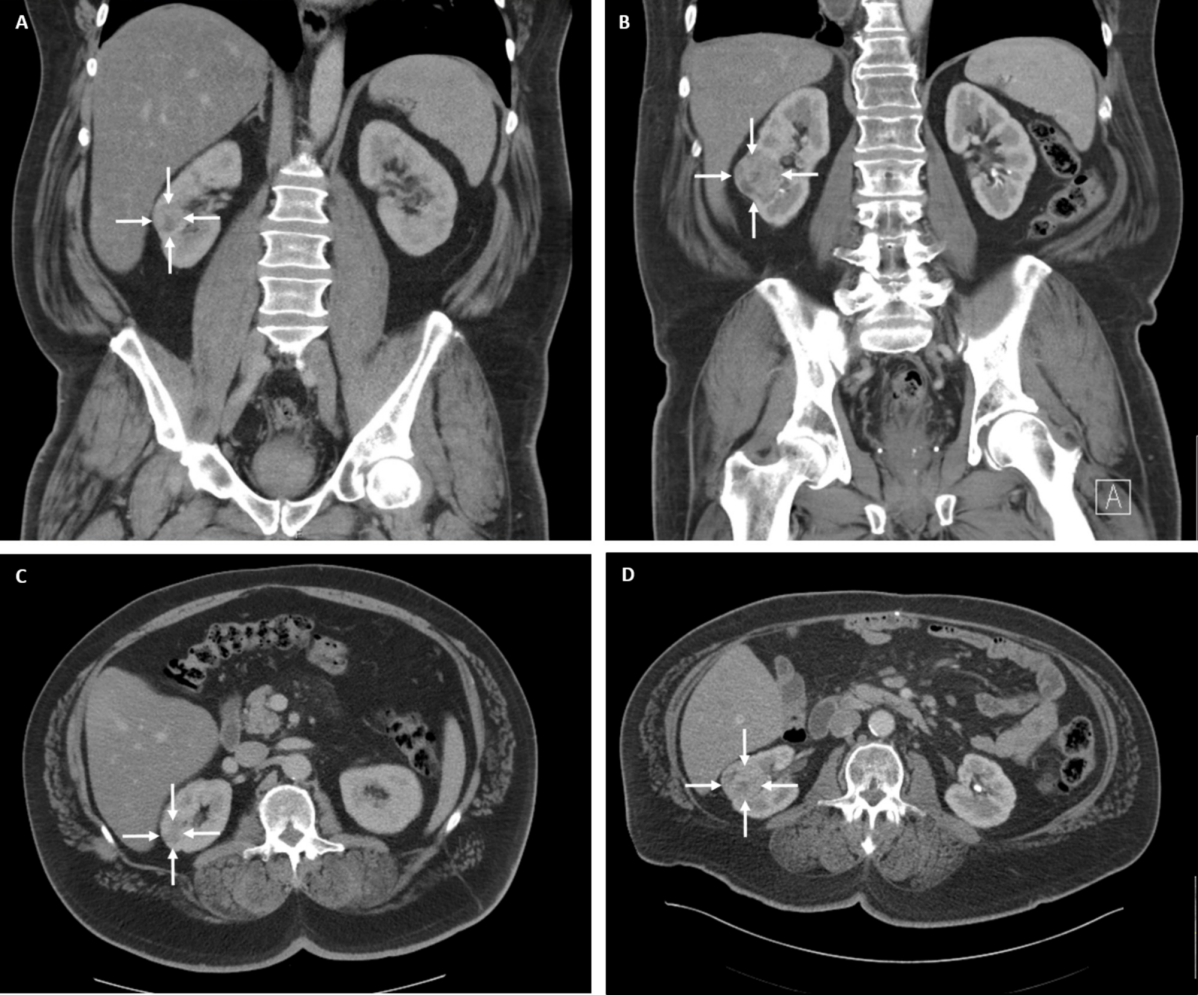
CT imaging of the right renal mass taken two years apart The initial CT coronal (A) and axial (C) views show a solid right renal mass measuring 2.7 cm. Two years later, coronal (B) and axial (D) views show the same solid right renal mass increased in size to 4.1 cm CT: computed tomography

A 62-year-old man had an incidental finding of a right renal mass on CT consistent with renal cell carcinoma (RCC). The patient had been under CT surveillance for his previous bowel cancer, and on review of imaging, the lesion was found to have been present for two years. During that time, it had increased from 2.7 to 4.1 cm (Figure 1).

He had normal renal function with a creatinine of 75 μmol/L and an estimated glomerular filtration rate greater than 90 mL/min/1.73m2. The patient had an extensive past medical history. He had undergone an open sigmoid colectomy and adjuvant chemotherapy for adenocarcinoma of the sigmoid (T3, N2, M0) five years ago. One-year prior, he had been treated with neoadjuvant chemotherapy followed by an Ivor Lewis esophagectomy for mucinous adenocarcinoma of the esophagus (T3, N1, M0). He was also on hormonal treatment with a luteinizing hormone-releasing hormone analog for Gleason 4+5 prostate cancer with a presenting prostate-specific antigen (PSA) of 59 ng/mL. His PSA level was stable on treatment with a reading of 0.34 ng/mL. In addition, he was a recurrent stone former and had been treated on multiple occasions with ureteroscopy and laser stone fragmentation.

The patient’s case was reviewed at a specialist urology multidisciplinary team meeting, and a decision was made to proceed with a radical nephrectomy. There were some concerns raised surrounding the feasibility of laparoscopic surgery, given his extensive previous abdominal surgeries. However, a decision was made to proceed with a laparoscopic right radical nephrectomy with plans to convert to open in case of technical difficulty.

An attempt made to insert a laparoscopic port, using Hasson's technique, was immediately met with difficulty due to adhesions. The procedure was converted to open using a loin approach, and a right radical nephrectomy was performed.

The patient was initially well but was found to have a rising C-reactive protein (CRP) level on postoperative day three, subsequently followed by spiking temperatures. He had an urgent CT scan, which suggested a bowel perforation with multiple abdominal and pelvic collections. The patient returned to the operating theater the same day for an emergency laparotomy. He was found to have two perforations of the small bowel consistent with injury from the insertion of a laparoscopic port. Small bowel resection and primary anastomosis were performed. Following this, he made a good recovery with no further complications and was soon discharged.

Postoperative histology of the kidney showed a G2 pT1a clear cell RCC with clear surgical margins. At a six-week follow-up, he remained well and pain-free. At two months, he presented with left loin pain and was found to have an 11-mm stone at the pelvi-ureteric junction in his solitary left kidney, causing an obstruction and an acute kidney injury. This was treated with emergency ureteric stent insertion followed by ureteroscopy and laser stone fragmentation.

Six months following his nephrectomy, the patient remains well and stone-free with no evidence of recurrence or metastatic disease. Given his history of multiple malignancies, he has also been referred for genetic assessment.

## Discussion

In this case, the patient was diagnosed with a small renal mass, and a decision was made to treat with a radical nephrectomy. Increasingly, patients with small renal masses are being treated with partial rather than radical nephrectomy. In appropriately selected patients, partial nephrectomy has the equivalent oncological outcomes with a reduced incidence of chronic kidney disease and non-cancer related mortality [6]. However, a partial nephrectomy is technically more challenging, requiring advanced laparoscopic skills to resect the tumor, secure hemostasis, and perform renal reconstruction promptly to minimize warm ischemic time. For instance, the British Association of Urological Surgeons (BAUS) nephrectomy audit has shown that partial nephrectomy is more commonly performed for tumors less than or equal to 4 cm in the UK. However, for tumors between 4 and 7 cm in size, radical nephrectomy is preferred [7].

In addition to tumor size, there are several other factors to consider in determining if a partial or radical nephrectomy is more suitable: the function of the contralateral kidney, whether the tumor is exophytic (protruding out) or endophytic (contained), the involvement of the collecting system, and the involvement of the renal hila (The R.E.N.A.L. nephrometry score) (Table 1).

**Table 1:** The R.E.N.A.L. nephrometry score-based assessment of renal tumor complexity R.E.N.A.L.: radius, exophytic/endophytic, nearness of the tumor to collecting system, anterior/posterior, location relative to polar lines

Tumor characteristics	
Radius (cm)	<4
	>4 and <7
	>7
Exophytic/endophytic	>50% exophytic
	<50% exophytic
	Entirely endophytic
Nearness to the collecting system or sinus (mm)	>7
	>4 and <7
	<4
Anterior/posterior	Anterior
	Posterior
	Neither
Location relative to polar lines	Entirely above or below
	Crosses a polar line
	>50% across polar line
	Crosses axial renal midline
	Entirely between polar lines
Hilar tumor	Yes
	No

Although not widely used in the UK, The R.E.N.A.L. nephrometry score was developed to assess the complexity of a tumor based on several of these factors [8]. Although there is no clear evidence as to what score would indicate a preference for radical nephrectomy, it can be a helpful tool when counseling a patient about their options. Using the R.E.N.A.L. nephrometry score in this case, the tumor would be classified as having intermediate complexity associated with a major complication rate for partial nephrectomy of 11.1% [9]. With the anticipated difficulties relating to the patient’s previous abdominal surgery, radical nephrectomy was the preferred option.

Previous abdominal surgery results in the formation of adhesions, abnormal fibrous bands between organs and/or the abdominal wall, in more than 90% of patients [10]. As in this case, adhesions can make laparoscopic surgery technically difficult and increase the risk of complications related to port-site placement. The impact of previous abdominal surgery in non-urological laparoscopic procedures has been extensively reviewed [11]. However, few studies have evaluated the outcomes of urological laparoscopic procedures such as nephrectomy.

Laparoscopic nephrectomy can be performed using a retroperitoneal or transperitoneal approach. The retroperitoneum is located anterior to the transversalis fascia and posterior to the parietal peritoneum. After accessing this space, the psoas muscle and posterior aspect of the kidney are first identified, followed by the renal hila, thereby allowing early vascular control [12]. The working space is increased with the flank position and balloon dilatation. However, despite these measures, the working space is significantly smaller than in the transperitoneal approach. There is also a scarcity of anatomical landmarks and considerable retroperitoneal fat that can make the retroperitoneal approach technically more challenging. In the transperitoneal approach, access is achieved via the anterior abdominal wall. The transperitoneal approach provides sufficient working space and familiar anatomical landmarks, but the colon must be mobilized to incise the posterior parietal peritoneum to access the kidney. Therefore, there will be an increased risk of intra-operative complications such as bowel injury [12].

Comparisons of the two approaches have consistently demonstrated a decreased operative time with a retroperitoneal approach (as there is no need to mobilize the colon using this approach) but no difference in overall oncological outcomes [13]. There are no guidelines as to which approach is preferable as both have their advantages and limitations. Generally, a surgeon’s experience and training will determine their approach to laparoscopic nephrectomy. In the UK, the BAUS nephrectomy audit showed a preference for the transperitoneal approach; this may due to the larger working space and familiar anatomical landmarks [14].

Historically, previous abdominal surgery was viewed as a relative contraindication to laparoscopy. It was thought to be technically difficult in addition to offering an increased risk of complications as a result of adhesions [15]. However, it is no longer deemed a contraindication with appropriate surgical training, planning, and technique. When considering patients with previous abdominal surgery for laparoscopic nephrectomy, it has been suggested that a retroperitoneal approach would prevent adhesion-related difficulties [16]. This is potentially supported by this case in which a transperitoneal approach was attempted and a bowel injury was encountered. However, other studies have demonstrated no significant difference between the two techniques in terms of technical difficulty, blood loss, or complication rate in patients with a history of abdominal surgery [17].

The site of the previous abdominal surgery may be a more significant factor. It is reasonable to consider that a right laparoscopic nephrectomy using a transperitoneal approach will be more difficult in a patient with a history of a previous open cholecystectomy than an appendicectomy, as the former may have caused more adhesions localized to the area of the kidney. Similarly, if previous intraperitoneal surgery could impact a transperitoneal approach, one could hypothesize that previous renal surgery would make a retroperitoneal approach technically more difficult. Only one study has explored the impact of previous open renal surgery or percutaneous nephrolithotomy in patients undergoing laparoscopic nephrectomy. The authors used a transperitoneal approach and found that laparoscopic nephrectomy could be performed safely and promptly in such patients [18]. However, no comparison was made to the retroperitoneal approach, which could be more difficult based on the location of the previous surgery.

These findings, although they are derived from small studies, indicate that there is currently no evidence to promote either a retroperitoneal or transperitoneal approach as a comparatively safer option in laparoscopic nephrectomy after previous surgery. A further evaluation, specifically based on the site of previous surgery, is required. To limit bias related to surgical experience, a large multi-institutional prospective study would be required. Such a study would also need to consider other factors that may influence the development of adhesions, such as abdominal radiotherapy and inflammatory conditions like endometriosis [10]. Also, comparisons are required between patients with a history of a single previous abdominal surgery and multiple surgeries. In this case, the patient had previously had two extensive surgeries, which may have increased the risk further. However, there is currently no evidence in the literature to endorse this.

The method of port placement is also an important consideration for patients with previous abdominal surgery. Port placement can be achieved using the Veress needle or open Hasson technique. The Veress needle involves blind port insertion as compared to the open Hasson technique in which the peritoneum is opened under direct visualization before placing the port into the intraperitoneal cavity. The safest technique for port insertion is a topic of great debate. The evidence suggests that, when performed by an appropriately trained surgeon, there is no significant difference in complication rate between the techniques [19]. However, in patients with a history of previous abdominal surgery, it is widely reported that there is an increased risk of complications if a Veress needle is used [20]. This is a logical finding given the likelihood of adhesions to the abdominal wall in such patients. Hence, an open Hasson technique must be recommended for patients with a history of previous abdominal surgery. Even in open Hasson techniques, the surgeon must be cautious of adhesions and the risk of bowel injury, as happened in this case. This highlights the importance of appropriate patient counseling specific to their risks.

## Conclusions

Our study examined a case of attempted transperitoneal right laparoscopic nephrectomy in a patient with a history of sigmoid colectomy and Ivor Lewis esophagectomy. The patient had significant adhesions and sustained a small bowel injury. The current evidence suggests that laparoscopic nephrectomy could be safely performed in patients with previous surgery but requires careful evaluation to identify specific risk factors such as the site of previous surgery. Also, an open Hasson technique should be recommended for laparoscopic port-site placement in such patients. Laparoscopic radical nephrectomy can be safely performed in these patients by using a multidisciplinary team approach that considers specific patient and tumor factors. However, as demonstrated in this case, patients are still at risk and must undergo appropriate preoperative counseling.
